# Microbiota profiles and antimicrobial resistance genes in sow fecal samples from farms with and without antibiotic use

**DOI:** 10.5713/ab.25.0062

**Published:** 2025-06-24

**Authors:** Sutthawongwadee Senawin, Chaiyapoom Bunchasak, Choawit Rakangthong, Chanwit Kaewtapee, Suporn Foongladda, Komwit Surachat, Rattapha Chinli, Wiriya Loongyai

**Affiliations:** 1 Department of Animal Science, Faculty of Agriculture, Kasetsart University, Bangkok, Thailand; 2 Department of Microbiology, Faculty of Medicine Siriraj Hospital, Mahidol University, Bangkok, Thailand; 3 Department of Biomedical Sciences and Biomedical Engineering, Faculty of Medicine, Prince of Songkla University, Songkhla, Thailand; 4 Translational Medicine Research Center, Faculty of Medicine, Prince of Songkla University, Songkhla, Thailand

**Keywords:** Antibiotic, Antimicrobial Resistance Genes, Gut Health, Gut Microbiota, Pig Farm, Sows

## Abstract

**Objective:**

Antibiotics have been used in swine production, and they are known to be associated with the gut microbiota and antimicrobial resistance (AMR). This study aimed to evaluate the dynamics of the microbiota and AMR among fecal bacteria in sowsby 16S rRNA gene sequencing and TaqMan array card assays.

**Methods:**

A total of 40 healthy multiparous sows were tested in a completely randomized design. Sows were randomly divided into two groups: one was fed a commercial diet with antibiotics for 3 weeks from mating to day 21 of gestation, before the farrowing stage (amoxycillin 300 mg/kg and tiamulin 150 mg/kg: control group, ABO), and the other was fed the same diet without antibiotics (treatment group, NOABO).

**Results:**

The ABO group had a higher alpha diversity than the NOABO group (p<0.05). The results re-vealed the highest bacterial abundance in the phylum Firmicutes in sow feces in the ABO group at an average level of 92.01% and 92.32% in the NOABO group. Erysipelotrichaceae, Clostridiaceae, and Terrisporobacter in the ABO group had enriched proportions. On the other hand, Lactobacillales, Bacilli, and Streptococcus were enriched in the NOABO group (p<0.05). In terms of AMR, a comparison of the normal log of resistance gene copies between the ABO and NOABO groups displays that the gene copy number was significantly higher (p<0.05) in the ABO group (59%) than in the NOABO group (41%) especially those of β-lactam, aminoglycosides, quinolones, and macrolides.

**Conclusion:**

Our investigations discovered that the core microbiota of withdrawal antibiotics may be related to the gut microbiota and AMR. Therefore, understanding the gut microbiota composition and function in animals could enable strategies for its modulation to improve sows’ gut microbiota and minimize the negative impact of antibiotics.

## INTRODUCTION

Thailand had a production volume of approximately 20.45 million swine in 2020, with one sow producing approximately 19.63 fattening pigs per year [1]. Therefore, sows are a very valuable role in swine production due to the influence of many economic factors. This is because the breed, diet, management and health directly affect the quantity and quality of piglets. In Thailand, reducing the sow replacement rate will decrease the replacement costs of economic losses due to sow weakness which was calculated in 2021 as worth more than 996.9 million baht, and increase sow productivity [1]. As a result, sows are kept in continuous production, where they are exposed to antimicrobials and varying populations of bacteria from several locations and animals for extended periods, which tend to increase antimicrobial resistance (AMR) [2]. Furthermore, sows’ increased nutritional intake to support the growth of the fetus and associated tissues, mammary development, endogenous heat production, and adaptive physiological processes result in a microbial imbalance, which induces a lower immune response, increased sensitivity to illness, AMR, and a loss in production performance [3]. During the late pregnancy and lactation stages, sows start suffering stress induced by the severe metabolic burden and do not fully recover until the weaning period. Stress in sows during this period decreases reproductive performance indicators, such as total litter size, live litter size, and litter weight gain [4]. Furthermore, decreased feed intake in sows during lactation leads to a prolonged negative energy balance, a worsening body condition, and reduced milk production [5].

Antibiotics or antimicrobial agents are a very valuable tool not only in human medicine but also in the treatment of livestock. The excessive or inappropriate use of antibiotics contributes significantly to the emergence and spread of AMR, AMR occurs when microorganisms change over time and no longer respond to antibiotics, making infections harder to treat and increasing the risk of disease spread, severe illness, and death [6]. Consistent with some countries prohibit antibiotics as feed additives in livestock, they are commonly used in many Southeast Asian countries such as Thailand, Malaysia, Vietnam and Indonesia [7,8]. This overuse of antibiotics in livestock is one likely driver of the high AMR burden and increased economic costs in Southeast Asia [9], including high rates of extended-spectrum β-lactamase (ESBL) and CTX-M enzymes [8]. AMR is a looming public health crisis at present; widespread resistance to antibiotics among bacteria is the cause of hundreds of thousands of deaths every year. AMR in food animals such as swine can impact human health via the direct introduction of AMR pathogens into the food chain by promoting the horizontal transfer of resistance determinants to other gut microbiota bacteria or pathogens [10]. A recent study has showed that the commensal microbiota in swine may become a reservoir of resistance to genes for pathogenic bacteria. This may contaminate meat destined for human consumption [11].

Recent reports indicated that the prevalence of antimicrobial resistant *Escherichia coli* is on the increase [12] and the infections caused by resistant bacteria usually fail to respond to treatment by specific antimicrobial agents [13]. Furthermore, antibiotics may contribute to or cause dysbiosis in the gut microbiota by directly eliminating the bacterial abundances and reducing the beneficial microbiota composition in sows. They could decrease the colonization of the *Lactobacillus*, *Bifidobacterium*, or *Prevotella* genus and promote harmful bacteria, such as *Clostridiums* spp. or *Escherichia coli* with a reduction in gut microbiota diversity [14]. Antibiotics can also disrupt the balance between the various species of the fecal microbiota. For example, by causing an increase in species richness, antibiotics can lead to the overgrowth of pathogenetic bacteria, such as toxigenic *Clostridioides difficile* in human [15]. Previous studies [15,16] in human found that at genus level, the antibiotic-treated group was characterized by a higher richness of *Shigella, Enterococcus*, *Bifidobacterium*, and *Bacteroides* and a lower abundance of *Lactobacillus* and *Allobaculum*. Moreover, administration of a combination of meropenem, gentamicin, and vancomycin resulted in an increase in the abundance of Enterobacteriaceae and other pathobionts, and a decrease in Bifidobacterium and butyrate-producing species in human [17], which had both short- and long-term negative health consequences [18]. Broadly speaking, infections caused by resistant bacterial strains lead to up to two-fold higher rates of adverse outcomes compared with similar infections caused by susceptible strains in human. These adverse outcomes may be clinical (death or treatment failure) or economic (costs of care, length of stay) and reflect both treatment delays and the failure of antibiotic treatment to cure infections [19]. For example, in the case of bacteremia and other serious infections due to methicillin resistant *Staphylococcus aureus*, a significantly higher case fatality rate has been clearly demonstrated as compared with methicillin-susceptible *S. aureus* infections [20]. Furthermore, among adults with bacteremic pneumococcal pneumonia, infection with penicillin-nonsusceptible pneumococci is associated with more than four times the risk of suppurative complications [21].

Therefore, the aim of this field study was to evaluate the dynamics of the microbiota and AMR among fecal bacteria in the commensal bacteria of sows. The fecal microbiota was characterized using data from 16S rRNA gene sequencing of the microbial community. We also assessed phenotypic AMR using a TaqMan array card. Our hypothesis is that the fecal microbiota composition and AMR genes in sow temporal dynamics are largely a function of antibiotic use. These studies demonstrated that stopping the use of antibiotics in feed has been associated with increased microbiota diversity and decreased abundance of certain antibiotic resistance and bacterial metabolic resistance genes.

## MATERIALS AND METHODS

### Animals and management

All experimental protocols were approved by the Institutional Animal Care and Use Committee of Kasetsart University, Thailand. Approval ID for Animal Care and Use for Scientific Research is ACKU66-AGR-025. A total of 40 healthy multiparous sows (Large white×Landrace, 2nd–5th parity), no disease or diarrhea occurred at least one week before sampling were tested in a completely randomized design. Sows were randomly divided into 2 groups in two different swine management systems including feed commercial diet with antibiotic for 3 weeks from mating to day 21 of gestation, before the farrowing stage (amoxycillin 300 mg/kg and tiamulin 150 mg/kg: control group, ABO) and same diet without antibiotic (treatment group, NOABO). Each experimental group consisted of two replicate pens, with 10 pigs housed in each pen (20 pigs per group). Sows were individually housed in farrowing crates (2.2 m×0.6 m) under an evaporative cooling system maintaining a temperature of 24°C–28°C and humidity of 70%–80%. Feed was manually supplied once daily, while water was provided ad libitum via nipple drinkers. Pens featured partially slatted floors for waste drainage. The house was cleaned weekly and feces were removed daily. Animal care complied with humane standards.

### Fecal sample collection

Sows were individually raised in different pens in the same house and 40 fecal samples were randomly selected from different pens. Fecal samples were collected by sterile 2 mL centrifuge tubes without any treatment, and these samples were used for 16s rDNA gene sequencing analysis and antimicrobial resistance TaqMan array card (AMR-TAC). Fecal samples were collected by plastic bags (approximately 300 g of each fecal sample). All the collected samples were kept cool in an ice box for transportation and then stored at −20°C in the laboratory before DNA extraction.

### DNA extraction, 16S rRNA amplification, and Illumina MiSeq sequencing

Microbial DNA was extracted by sampling 300 mg of feces from each sample. Genomic DNA was isolated by adding bead-beating using a Qiagen Power Fecal DNA Kit (Qiagen) following the manufacturer’s instructions. Zirconium glass beads (400 mg; 0.1 mm diameter, BioSpec Products) were added to the extraction system followed by vigorous vortexing (twice) using a FastPrep-24 Instrument (MP Biomedicals) at a speed of 6.0 m/s for 1.5 minutes. Extracted DNA was confirmed using agarose gel electrophoresis.

The 341F (5′-ACTCCTACGGGAGGCAGCAG-3′) forward primer and 806R (5′-GGACTACHVGGGTWTCTAAT- 3′) reverse primer was used to amplify the V3–V4 hyper-variable region of the 16S rRNA gene. PCR conditions were pre-denaturation at 94°C for 4 min, denaturation process at 94°C for 30 s, annealing process at 50°C for 45 s, and elongation process at 72°C for 30 s for 25 cycles. Finally, the extension process was done at 72°C for 5 min. The PCR product was purified and used to construct a library, and then, paired-end sequencing (2×250) was performed on a MiSeq platform (Illumina) from Suzhou GENEWIZ Biological Technology.

### Sequencing and phylogenetic analysis

To obtain accurate and reliable results during bioinformatics analysis, the Quantitative Insights into Microbial Ecology (QIIME) software program (v2022.2) was used to filter raw data. The fragments were then clipped and deleted to a <25 mass score and >225 bp length, respectively. Low-quality sequences were removed, and the remaining sequences were processed and analyzed using the QIIME (v2022.2) software. The sequencing data were clustered into operational taxonomic units (OTUs) at 97% similarity using standard procedures, and chimeric or erroneous sequences were removed from the optimized dataset. Alpha diversity was assessed using the Shannon index (diversity) and observed OTUs (richness). These analyses and rarefaction curve plotting were performed using the phyloseq package in R (v3.0.3). Beta diversity was evaluated using Bray–Curtis, weighted UniFrac, and unweighted UniFrac distances with principal coordinate analysis (PCoA), conducted via QIIME software (v2022.2). The machine learning method random forest was used to identify the top 29 genera and their functions. Further phylogenetic trees of the species-level genome bins were built by QIIME2 (v.2.1.10) and visualized using iTOL (v.6.6). The phylogenetic analysis was carried out by subjecting raw sequencing reads to analysis using MetaPhlAn V1.7.8 [22] incorporating BowTie2 [23]. Raw reads were also uploaded to the MG-RAST pipeline [24] for functional and taxonomical assignment along with estimation of taxonomic abundance. The products were analyzed on a model ABI PRISM 3700 DNA analyzer system (Applied Biosystems). Nucleotide sequences were analyzed with GENETYX-Mac Version 11.2 software (Software Development), FASTA search, and a Chimera Check program of the Ribosomal Database Project.

### Antimicrobial resistance TaqMan array card

AMR-TAC from Applied Biosystems, a brand of Thermo Fisher Scientific was utilized as previously described [25]. Briefly, TaqMan probe oligonucleotides and primer were synthesized and spotted on to the microfluidic card. 20 μL of input DNA extracts from fecal DNA extraction process were mixed with 50 μL of 2× PCR buffer, 4 μL of 25× PCR enzyme of AgPath-ID-PCR kit (Applied Biosystems, Life Technologies), and 26 mL of nuclease free water to yield a 100 μL final volume. This mixture was loaded into each port of the card, and the card was centrifuged twice at 1,200 rpm (approximately 105×g, rotor radius 6.5 cm) for 1 min each and then sealed. The loading ports were excised, and the full card was inserted into a ViiA 7 instrument (Thermo Fisher Scientific). Cycling conditions included an initial denaturation process at 95°C for 10 min, followed by 40 cycles of denaturation process at 95°C for 15 s and extension processes at 60°C for 1 min.

### Evaluation of standard curve and normalization

The TaqMan array card was performed as previously described [25]. Synthetic positive control plasmids (Genewiz) which contained primer/probe regions of all targets were 10-fold serially diluted in 5×10^7^ to 5 copy/μL. 20 μL of each diluted sample was tested in triplicate by mixing with PCR reagents to a total volume of 100 mL then loaded into the array card. The final concentration ranged from 1×10^7^ to 1 copy/reaction. The average cycle threshold (Ct) value was used to generate standard curve for later quantification and conversion of Ct values to gene copy number of fecal samples. The Ct value was converted to copy number by using standard curves for each target and then the resistance gene copy number was normalized to 10^6^ bacterial 16S rRNA gene copy number of each sample. These results copy number of resistance genes per 10^6^ bacterial 16S rRNA gene copies, which allowed comparisons between fecal samples. The normalized gene copy number was used for analysis and when binary (positive/negative) results were required, the gene copy number cut-off was used whereby ≥1 gene copy was positive.

### Statistical analysis

Alpha-(α) diversity Shannon (evenness), Observed feature, Faith’s phylogenetic diversity and Pielou’s index were calculated with the R phyloseq package. Shannon indices between ABO and NOABO group were statistically compared with Kruskal-Wallis test, using Excel software statcel2. Beta-(β) diversity was estimated using un-weighted UniFrac distances and tested by a PCoA, using the R phyloseq package. The UniFrac distance between groups was statistically analyzed by permutational multivariate analysis of variance (PERMANOVA), using QIIME2 program. The relative abundances (%) of all detected bacterial genera between groups ABO and NOABO were calculated and statistically compared with the Kruskal-Wallis test. Linear discriminant analysis (LDA) effect size (LEfSe) was used to determine significant differences in abundance among different sample groups [26] and a logarithmic LDA score≥2.0 was used as a threshold. The principal component analysis and hierarchical clustering were generated using ClustVis [27]. The singular value decomposition with imputation was used to calculate principal components and Euclidean distance and average linkage were used for clustering. We used a PERMANOVA test to test differences in AMR genes between antimicrobial use groups by using the Adonis function of the vegan package in R version 3.6.3.

## RESULTS

### Alpha and beta diversity

**Figure 1:**
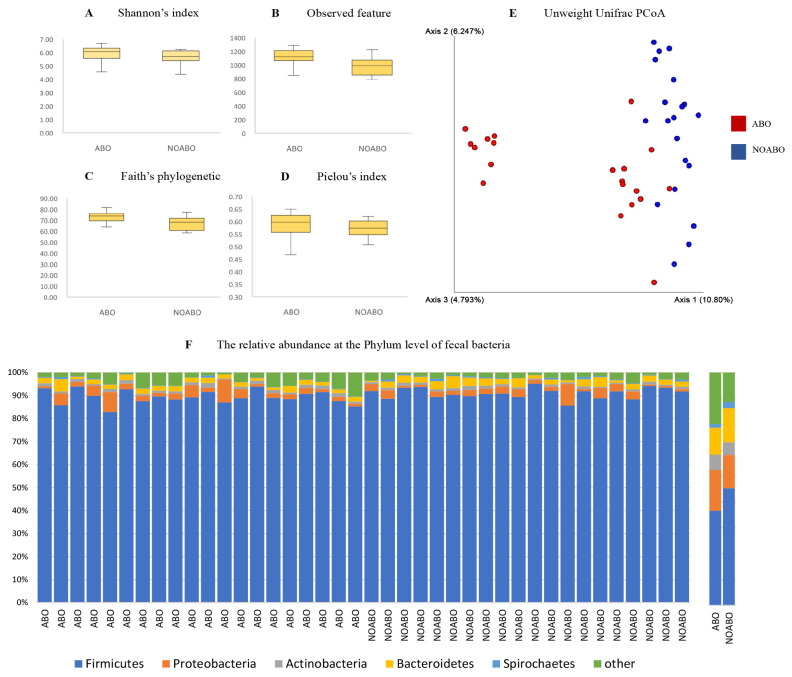
Diversity indices and fecal bacterial composition in ABO and NOABO groups. (A–D) Alpha diversity indices including Shannon’s index, observed features, Faith’s phylogenetic diversity, and Pielou’s evenness are shown as boxplots. (E) PCoA based on unweighted UniFrac distance shows clear separation between ABO (red) and NOABO (blue) groups. (F) Relative abundance of fecal bacteria at the phylum level, with Firmicutes (blue), Proteobacteria (orange), Actinobacteria (gray), Bacteroidetes (yellow), Spirochaetes (red), and others (green). Data were analyzed using QIIME2. ABO: antibiotic-treated; NOABO: control group. PCoA, principal coordinate analysis; QIIME, Quantitative Insights into Microbial Ecology.

Bacterial biodiversity in sow manure was studied in the antibiotics (ABO) and non-antibiotics (NOABO) groups for 3 weeks from mating to day 21 of gestation, before the farrowing stage. Alpha diversity analysis was conducted using four indices: Shannon’s index, observed features, Faith’s phylogenetic diversity, and Pielou’s index (Figures 1A–1D). The ABO group exhibited significantly higher alpha diversity than the NOABO group, as indicated by Shannon’s index (p = 0.048), observed features (p = 0.002), and Faith’s phylogenetic diversity (p = 0.001). However, no significant difference was observed in Pielou’s index between the two groups (p = 0.144). The beta diversity showed overlapping and scattering of microbial communities (Figure 1E), which is also supported by non-metric multidimensional scaling plots (NMDS) between the ABO and NOABO groups. Furthermore, NMDS showed that the fecal microbiota of the ABO group was distinct from that of the NOABO group (Figure 1E). PCoA in both groups presented distinguished and separated clusters showing a profound difference in microbiota diversity (p = 0.001) between different animal groups. Using the unweighted unifrac distance, the cluster of eight samples from the ABO group was regrouped together and separated from the other clusters of the ABO and NOABO groups that were clustered close.

### Microbial composition at different taxonomic level

The QIIME 2 program was used to perform a quality check, and subsequent analysis of the bacterial data showed that approximately 224,450 to 358,654 reads were present (total frequency = 10,703,703). The bacterial genera present in sow feces (relative abundance) were compared by analysis at the phylum level by the LEfSe method (LDA LEfSe) to show the percentage of bacteria that were statistically significantly different at the 95% confidence level. At the phylum level, several bacterial phylum with relative abundances greater than 0.01% were identified in fecal samples. However, only the dominant phylum are presented in Figure 1F for clarity. The results revealed that the phylum of greatest abundance in sow feces was Firmicutes in sow feces at an average level of 92.01% and 92.32% in the ABO group and NOABO group, respectively. Proteobacteria, Actinobacteria, and Bacteroidetes were found at 3.24% and 2.6%, 1.23% and 1%, 2.15% and 2.67% in the ABO and NOABO groups, respectively.

**Figure 2:**
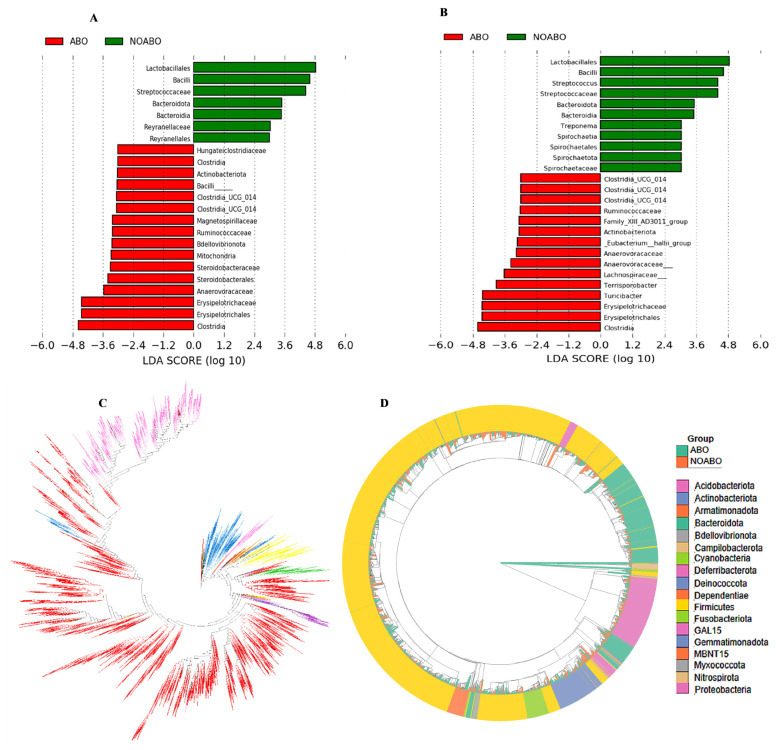
Differential bacterial taxa and phylogenetic structure of gut microbiota between ABO and NOABO groups. (A–B) Linear discriminant analysis effect size (LEfSe) identifying taxa significantly enriched in ABO (red) and NOABO (green) groups at the family (A) and genus (B) levels. LDA score >2.0, p<0.05. (C) Cladogram showing taxonomic hierarchy of significantly different taxa between groups, colored by the group in which they are enriched. (D) Circular phylogenetic tree of all detected taxa, highlighting ABO and NOABO groups with distinct inner rings. Outer ring colors represent bacterial phyla. ABO: antibiotic-treated group; NOABO: control group. LDA, linear discriminant analysis.

LEfSe analysis was performed to identify differentially abundant bacterial taxa between the ABO and NOABO groups. At the family level (Figure 2A), Lactobacillaceae, Streptococcaceae, Bacteroidaceae, and Reyranellaceae were significantly enriched in the NOABO group (p<0.05), while members of Ruminococcaceae, Erysipelotrichaceae, Anaerovoracaceae, and several Clostridia-related families were enriched in the ABO group. At the genus level (Figure 2B), *Lactobacillus, Streptococcus, Bacteroides, Treponema*, and *Spirochaeta* were more abundant in the NOABO group, whereas *Clostridium_UCG_014, Eubacterium hallii group, Turicibacter*, and *Anaerovorax* were enriched in the ABO group. These findings indicate distinct microbial profiles between the two groups.

To compare the phylum-level diversity on the phylogenetic tree of bacteria detected with the V3–V4 targeted primer pair versions, we selected phyla that were detected with at least one primer pair version in at least one sample with more than 0.1% relative abundance. Phyla detected at a lower relative abundance were excluded from these results (Figure 2C). A phylogenetic tree was constructed to explore the evolutionary characteristics of diversity in the gut microbiota. Firmicutes was the most widely distributed phylum throughout the phylogenetic tree, with taxa spanning across multiple branches and clades, reflecting high taxonomic diversity. Bacteroidetes also showed broad distribution, particularly across both ABO and NOABO groups. In contrast, Proteobacteria appeared predominantly within the NOABO group but was observed in several distinct branches as well (Figure 2D).

### Antimicrobial resistance gene analysis

**Figure 3:**
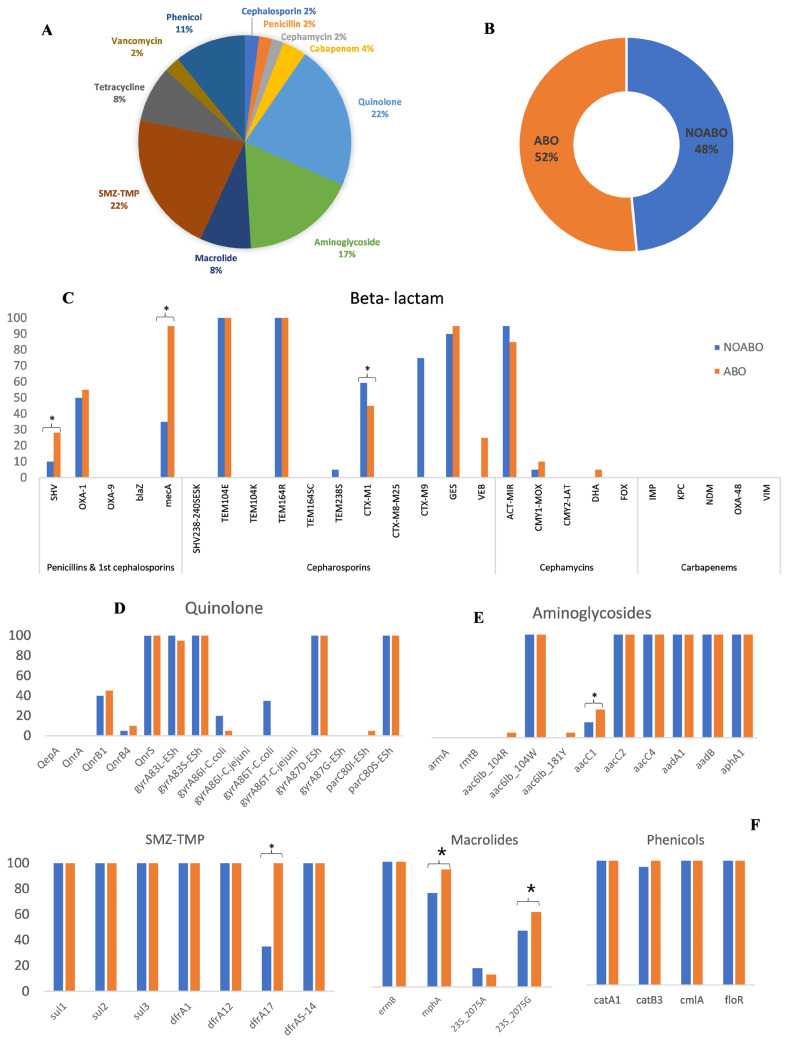
Distribution and prevalence of antimicrobial resistance (AMR) genes in ABO and NOABO groups. (A) Proportion of resistance-associated gene copies across antibiotic classes, expressed as a percentage of normalized gene copies per million 16S rDNA copies. (B) Overall prevalence of AMR genes in the ABO and NOABO groups. A gene was considered positive when copy number ≥1. (C–F) Prevalence of resistance-associated genes categorized by antibiotic class: beta-lactam (C), quinolones (D), aminoglycosides (E), and macrolides, sulfonamides (SMZ-TMP), and phenicols (F). Orange bars represent ABO (antibiotic-treated) and blue bars represent NOABO (control groups). Asterisks (*) indicate statistically significant differences between groups (p<0.05).

We examined the gene copy numbers to assess the proportions of the different AMR genes present. Among the genes detected for resistance to antibiotic groups, those encoding resistance to SMZ-TMP (22%) and fluoroquinolone (22%) were present in the highest proportions (Figure 3A). Furthermore, a percentage comparison found that the normalized gene copy per million 16s rDNA copies was significantly higher in the ABO vs the NOABO group (52% vs 48%; p< 0.05, Kruskal-Wallis H test) (Figure 3B).

In addition to gene copy numbers, we examined the positivity rate of detected AMR genes between groups. The positivity rate of β-lactam resistance genes in both groups was 13/26 (50%). Notably, several AMR genes exhibited a higher detection rate in the ABO group, including *SHV*, *GES*, and *OXA-1* for β-lactam (Figure 3C); *QnrB1, QnrB4*, and *parC80I-Esh* for fluoroquinolones (Figure 3D); *aac6Ib_104R and aacC1* for aminoglycosides (Figure 3E); *dfrA17* for sulfa-trimethoprim. Although *mphA*, *23S_2075A*, and *catB3* were detected more frequently in the ABO group, these differences were not statistically significant (p>0.05).

A total of 13 genes (50%) were not detected in any samples. These included *IMP*, *KPC*, *NDM*, *OXA-48*, *VIM*, and *DHA* for β-lactams (Figure 3C); *QepA* and *QnrA* for fluoroquinolones (Figure 3D); and *armA* and *rmtB* for aminoglycosides (Figure 3E). Twenty-two genes showed similar prevalence between the two groups (no statistical difference), including *TEM104E* and *TEM164R* (Figure 3C); *QnrS*, *gyrA83L-Esh*, *gyrA83S-Esh*, *gyrA87D-Esh*, and *parC80S-Esh* (Figure 3D); *aac6Ib_104W*, *aacC2*, *aacC4*, *aadA1*, *aadB*, *aphA1* (Figure 3E); *sul1*, *sul2*, *sul3*, *dfrA1*, *dfrA12*, *dfrA5-14*, *ermB*, *catA1*, *cmlA*, and *floR* (Figure 3F). Additionally, 26 resistance genes were highly prevalent (91%–100%) in both groups. These included *TEM104E*, *TEM164R*, *GES*, and *ACT-MIR* (Figure 3C); *QnrS*, *gyrA83L-Esh*, *gyrA83S-Esh*, *gyrA87D-Esh*, and *parC80S-Esh* (Figure 3D); *aac6Ib_104W*, *aacC2*, *aacC4*, *aadA1*, *aadB*, *aphA1* (Figure 3E); and *sul1*, *sul2*, *sul3*, *dfrA1*, *dfrA12*, *dfrA5-14*, *ermB*, *catA1*, *catB3*, *cmlA*, and *floR* (Figure 3F).

## DISCUSSION

Antibiotics are widely used in human medicine and animal production, making important contributions to human health and animal husbandry development [28]. Today, however, the potential significance of the damaging effects of antibiotics on the gut microbiota has become a high-concern topic. Each use of antibiotics creates evolutionary pressure, in both human and veterinary medicine, leading to the emergence of resistance, which poses a significant threat to public health [29].

In this study, we withdrew amoxicillin and tiamulin for 3 weeks during the post-partum or farrowing stage in the NOABO group, and antibiotics were continued during the post-partum period in the ABO group. The alpha diversity analysis revealed significantly higher microbial diversity in the ABO group than in the NOABO group, as indicated by Shannon’s index, observed features, and Faith’s phylogenetic diversity. These findings suggest greater richness and phylogenetic variability in the ABO group. In contrast, no significant difference in Pielou’s index was observed, indicating that the distribution of taxa was comparable between groups despite differences in diversity levels. It should be noted that reduced diversity does not necessarily mean a reduced number of bacteria overall [15]. In cases where the overall number of bacteria increases, it may be due to the elimination of antibiotic-susceptible bacteria and multiplication of antibiotic-resistant bacteria that take their place [10]. Consistent with the finding of Panda et al [30], who reported the broad-spectrum nature of intensive antibiotic treatment, full eradication of gut microbiota was not achieved, and numerous species remained detectable by Day 4 (D4) after treatment antibiotic. This observation was based on a human clinical trial. By Day 8 (D8) post-treatment, microbial richness was still reduced; however, Shannon’s diversity index had increased significantly, suggesting that the surviving microbial populations had begun to recover through more balanced regrowth. In the present study, although the species diversity increased following antibiotic treatment, the total microbial load tended to decrease. This result differs from a previous study in which broad-spectrum beta-lactam antibiotics administered over a 7-day period led to a two-fold increase in both microbial load and diversity in human fecal samples [31]. Such divergence may be attributed to species-specific or host-specific microbiota responses, as well as differences in antibiotic types, dosage, or sampling intervals. Furthermore, our beta diversity analysis revealed disrupted microbial community structure following the administration of amoxicillin and tiamulin, suggesting a disturbance in microbiota homeostasis during the gestation period of sows. This period is particularly sensitive due to the critical role of maternal gut microbiota in nutrient metabolism, immune modulation, and fetal development [32]. Alteration of gut microbiota during this stage may thus have downstream consequences on reproductive efficiency and offspring health. These findings underscore the importance of cautious antibiotic use and the potential role of microbiota-supportive strategies, such as probiotic supplementation, especially during physiologically vulnerable periods in breeding animals [33]. The PCoA plot revealed a discrete separation of the overall bacterial community structure. Initially, before antibiotic cessations, the clustering structure of the community in both groups was expected to have the same pattern structure. After antibiotic withdrawal for 3 weeks, we found changes in the aggregation of structures in both groups, as if we had altered or disrupted the microbiota homeostasis in the sow’s gut. The resulting more pronounced PCoA expression in the NOABO group shows a profound difference in microbiota diversity. This suggests that the gut microbial community in animals not receiving antibiotic treatment (NOABO) remained more heterogeneous and stable, as evidenced by the wider distribution of sample points across the PCoA axes. In contrast, the ABO group displayed tighter clustering, particularly along axes 2 and 3 (Figure 1E), indicating a shift in microbial composition and community structure, likely resulting from the suppressive effects of antibiotics on a broad range of bacterial taxa. This directional segregation in the ABO group reflects specific microbial responses to antibiotic pressure that may not be fully captured by variation along axis 1, the clustering pattern also implies a degree of convergence in microbial community profiles, possibly due to the depletion of antibiotic-sensitive taxa and subsequent dominance of resistant strains. These observations are consistent with previous findings that antibiotic exposure leads to decreased gut microbial variability and increased risk of dysbiosis [32]. Furthermore, this result is similar to a previous research finding [34], which indicated that beyond antibiotic exposure itself, both the timing and type of antibiotics used were critical parameters influencing gene expression patterns in ABO and NOABO groups. These factors may help explain the degree of microbial community shift and spatial segregation observed in our study. At all-time points, the expression patterns from piglets of sows in the antibiotic withdrawal were separated from those of piglets from amoxicillin-treated sows. On day 7 however, all samples clustered closely together, confirming the lack of differentially expressed genes at the initial time point.

A substantial amount of gut microbiota sequence data was obtained from pregnant sows in this study, with read counts exceeding those typically reported in previous studies in sows. Although Reference [35] investigated non-pregnant sows under different physiological conditions, it is cited here solely to indicate the relative sequencing depth achieved, rather than for direct biological comparison. As a result, the microbiota analysis results in this study are more reliable. In our study, Firmicutes, Proteobacteria, and Bacteroidetes were the most abundant phylum, and significant differences were noted between the two groups. The relative abundance results were consistent with the results of phylogenetic tree analysis. In our research, antibiotics were continued during the post-partum period in the ABO group, resulting in an altered or disrupted gut microbiota in the sow. It appeared that in the ABO group, there was a significant increase in the abundance of *Clostridia*, which are classified in Clostridiaceae. *Clostridia* is a large genus of obligate anaerobes belonging to Firmicutes, as a predominant cluster of commensal bacteria in the gut, exerting many salutary effects on our intestinal homeostasis [36]. To date, *Clostridia* genus has been reported to attenuate inflammation and allergic diseases effectively owing to their distinctive biological activities [36]. Erysipelotrichaceae is a family within Firmicutes, commonly found in the intestinal tract of mammals, and has been associated with host metabolic disorders and inflammatory diseases [37]. One member of this family, *Erysipelothrix rhusiopathiae* is a facultative intracellular pathogen that is best known to cause erysipelas in all stages of pig production, both in piglets and in sows [38], presenting in acute, subacute, or chronic forms. In acute cases, pigs may die suddenly without symptoms, while subacute cases present with fever, stiff gait, and diamond-shaped skin lesions. Chronic infections often result in arthritis and endocarditis [39]. *Turicibacter*, another genus under Firmicutes, is a well-recognized commensal bacterium in the mammalian gut. It is linked to host metabolic traits such as dietary fat responses and fiber digestion, particularly through its association with butyric acid production and enhanced acid detergent fiber digestibility [35]. *Turicibacter* may play some positive roles in swine microbiome immune interactions, consequently promoting enhanced growth performance [40]. In this study, *Turicibacter* was significantly increased in the NOABO group, suggesting a potential compensatory mechanism that supports fiber fermentation and immune modulation in the absence of antibiotic exposure. In contrast, the presence of Erysipelotrichaceae members may reflect microbial imbalance or stress-induced pathogen proliferation. These findings highlight the dual functional roles of Firmicutes members, encompassing both beneficial commensals and opportunistic pathogens, which may differentially influence host health under varying physiological and treatment conditions. Whereas in the NOABO group, it turned out that *Lactobacillus* abundance was significantly increased. *Lactobacillus* belongs to the phylum Firmicutes and the family Lactobacillaceae, which are the most important probiotic bacteria. *Lactobacillus* spp. acts by regulating the luminal pH, enhancing barrier function by increasing mucus production, stimulating the secretion of antimicrobial peptides, and changing the gut microbial composition [41]. In a previous study, *Lactobacillus plantarum CAM6* in sows increased the content of lactose, nonfat solids, and mineral salts and the density of sows’ milk while decreasing milk fat. Moreover, the probiotic, when feed orally to the sows, improved their body weight and reduced the incidence of diarrhea in their offspring [42]. The results of this study indicate that the withdrawal of antibiotics during a 3-weeks period resulted in an increase in the abundance of the beneficial gut microbiota, especially that of *Lactobacillus*, as seen in the LEfSe analysis.

As the above results show, the impact of antibiotics on the gut microbiota is complex and variable, based on multiple factors such as sow characteristics, the type of antibiotic, and the antibiotic period [30]. Most prior studies evaluated short-term (<1–2 weeks) treatment with macrolides and its implications on the gut microbiota [43]. A recent meta-analysis summarized various clinical trials and concluded that short-term exposure to lincomycin was associated with significantly reduced alpha diversity in the gut microbiota of swine [44]. In this study, we found that amoxycillin and tiamulin treatment altered microbial diversity and the four core phyla, Firmicutes, Bacteroidetes, Proteobacteria, and Actinobacteria, consistent with previous research that the dominant phyla in the sow gut are Firmicutes, Bacteroidetes, Actinobacteria, and Proteobacteria [45]. Finally, the ABO group exhibited significantly higher alpha diversity indices than the NOABO group. In addition, the observed differences in bacterial abundance suggest that exposure to amoxicillin and tiamulin may have disrupted the gut microbiota environment in sows. While the present study focused on sows, previous reports in piglets also demonstrated that amoxicillin administration reduced microbial diversity and shifted bacterial composition, including an increase in Proteobacteria and a decrease in Firmicutes [45,46]. Although these findings provide general insights into the effects of antibiotics on gut microbiota, caution should be taken in direct comparisons due to physiological and microbial differences between sows and piglets. The use of antibiotics also has another impact on antibiotic resistance. In this study, we also detected AMR genes directly from fecal specimens among two different sow management systems. The antibiotic use group, which utilized amoxicillin and tiamulin in feed, were distinct and had a higher prevalence of AMR genes than the no antibiotic use group. The common β-lactamase (BL) genes observed in our study included *CTX-M1, OXA-1, ACT-MIR*, and *VEB*. Although *CTX-M1* has been reported as the most prevalent BL gene type in sow farms, and *CTX-M1* are common in Thailand, the high occurrence of *OXA-1* and *VEB* has not been reported by the Thai agriculture industry [16]. The ABO group showed a higher gene copy than the NOABO group, especially resistance genes to β-lactam, aminoglycosides, quinolones, and macrolides. One possible course for this incidence could be co-selection or co-transfer of gene cassettes on integrons [18]. Unexpectedly, the NOABO group showed a higher relative abundance of the *CTX-M1* gene, which encodes ESBL. This finding may be explained by the persistence of resistant strains in the absence of recent antibiotic pressure. Resistance genes such as *CTX-M1*-type β-lactamases are often carried on mobile genetic elements, which can be maintained within microbial communities even without antimicrobial exposure [47]. Furthermore, the reduced microbial diversity observed in the NOABO group may have allowed expansion of certain resistant taxa, as lower diversity has been associated with reduced colonization resistance and increased dominance of specific microbial populations [17]. These findings highlight that AMR may persist due to ecological factors beyond direct antibiotic use.

For macrolides, the erm gene was common and widespread [20]. However, the *mphA* gene was increased in the ABO group, which could be concerning since the *mphA*, a phosphotransferase that inactivates macrolides, is commonly found on bacterial mobile genetic elements and could have been induced by tylosin in sows. The *A2075G* gene is the most common mutation conferring macrolide resistance and was seen in this study at 67%.

We determined that larger scale, ideally quantitative, surveillance of human, environmental, and animal sources for AMR genes would be helpful to comprehend where the largest burden of AMR genes originates. Our study shows that direct molecular detection of AMR genes by Taq man Array Card through this approach is promising, and points to non-use of antibiotics in food producing animals, especially swine, as an important component of AMR, particularly β-lactamase resistance.

## CONCLUSION

In this study, the absence of antibiotic supplementation (amoxicillin 300 mg/kg+tiamulin 150 mg/kg) as prophylaxis in sow production decreased the abundance of pathogenic bacteria, increased microbiota diversity, and decreased the expression of certain antibiotic resistance genes. This suggests that antibiotic supplementation is negatively implicated in sow health, which could lead to an improvement in medical treatment strategies in order to avoid failure of antibiotic therapy. Therefore, this research contributes a novel resource and information with positive implications in health and disease prevention for sow industry.
